# A Novel NAD Signaling Mechanism in Axon Degeneration and its Relationship to Innate Immunity

**DOI:** 10.3389/fmolb.2021.703532

**Published:** 2021-07-08

**Authors:** Eleanor L. Hopkins, Weixi Gu, Bostjan Kobe, Michael P. Coleman

**Affiliations:** ^1^ John van Geest Centre for Brain Repair, Department of Clinical Neurosciences, University of Cambridge, Cambridge, United Kingdom; ^2^ School of Chemistry and Molecular Biosciences, Institute for Molecular Bioscience and Australian Infectious Diseases Research Centre, The University of Queensland, Brisbane, QLD, Australia

**Keywords:** NAD, NMNAT2, Sarm1, axon degeneration, innate immunity

## Abstract

Axon degeneration represents a pathological feature of many neurodegenerative diseases, including Alzheimer’s disease and Parkinson’s disease where axons die before the neuronal soma, and axonopathies, such as Charcot-Marie-Tooth disease and hereditary spastic paraplegia. Over the last two decades, it has slowly emerged that a central signaling pathway forms the basis of this process in many circumstances. This is an axonal NAD-related signaling mechanism mainly regulated by the two key proteins with opposing roles: the NAD-synthesizing enzyme NMNAT2, and SARM1, a protein with NADase and related activities. The crosstalk between the axon survival factor NMNAT2 and pro-degenerative factor SARM1 has been extensively characterized and plays an essential role in maintaining the axon integrity. This pathway can be activated in necroptosis and in genetic, toxic or metabolic disorders, physical injury and neuroinflammation, all leading to axon pathology. SARM1 is also known to be involved in regulating innate immunity, potentially linking axon degeneration to the response to pathogens and intercellular signaling. Understanding this NAD-related signaling mechanism enhances our understanding of the process of axon degeneration and enables a path to the development of drugs for a wide range of neurodegenerative diseases.

## Introduction

Neurons are the longest cells in the body; this structure lends itself to their function of transmitting signals around the body to react to changing stimuli, but their length leaves them vulnerable to injury and other stresses. As they cannot rely solely on diffusion of cell survival factors over distances up to 1 m, they have to actively transport a multitude of molecules along their axons in an energy-intensive process (De Vos et al., 2008). Changes in metabolism can disrupt axon transport and lead to degeneration, as can physical injury such as axon transection or crush (Coleman, 2005). Chemicals such as vincristine and paclitaxel are also known to induce degeneration by inhibiting axonal transport, limiting their use as cancer chemotherapeutics (Lapointe et al., 2013; Turkiew et al., 2017; Geisler et al., 2019a). Recently, it has been shown that neuroinflammation and necroptosis can also induce axon degeneration (Ko et al., 2020).

The first stage of many neurodegenerative diseases is axon degeneration, precisely because of their vulnerability to damage (Stokin et al., 2005; Cheng et al., 2010; Loring and Thompson, 2020). Diseases involving axon loss, such as Alzheimer’s disease (AD), amyotrophic lateral sclerosis (ALS) (van Rheenen et al., 2016) and peripheral neuropathies, contribute greatly to global disease burden, and are examples of such diseases where axon degeneration is involved in disease progression (Stokin et al., 2005; Geisler et al., 2016; Coleman and Höke, 2020).

We review here the well-understood and widely-occurring mechanism, programmed axon degeneration (or Wallerian degeneration), in which NAD (nicotinamide adenine dinucleotide) and related biology are known to play a central role. A large number of disease models in animals and cell culture, and a growing list of human disorders involve axon loss through this mechanism (Conforti et al., 2014; Coleman and Höke, 2020; Loreto et al., 2020b).

NAD is a molecule found in all the kingdoms of life. It exists in either oxidized (NAD^+^, electron acceptor) or reduced form (NADH, electron donor). Under physiological conditions, the cytoplasmic NAD^+^-to-NADH ratio is from 1 to 700, while in mitochondria, this ratio is maintained at 7–8 (Ying, 2008). In cells, NAD is dynamically regulated through continuous biosynthesis, consumption and recycling. In mammals, NAD is synthesized from three main pathways. The *de novo*, Preiss-Handler and salvage pathways use tryptophan, nicotinic acid (NA) and nicotinamide (Nam), respectively to generate NAD. NAD-consuming enzymes include sirtuins, PARPs (poly(ADP–ribose) polymerases), CD38 (cluster of differention 38) (Navarro et al., 2021) and SARM1 (sterile alpha and Toll/interleukin-1 receptor motif-containing 1) (Essuman et al., 2017). NAD is recognized for its ability to carry electrons in redox reactions (Warburg and Christian, 1936; Nikiforov et al., 2015), and plays an important role in various physiological processes including energy metabolism, DNA repair and transcriptional regulation (Ziegler and Oei, 2001; Luo and Kraus, 2012; Cantó et al., 2015), and in pathological processes associated with neurodegeneration, cancer and inflammation (Demarest et al., 2019; Lautrup et al., 2019). Emerging evidence reveals that NAD depletion is found during mitochondrial dysfunction, impaired DNA repair and inflammation, while an NAD increase enhances metabolic fitness (Lautrup et al., 2019).

**FIGURE 1:**
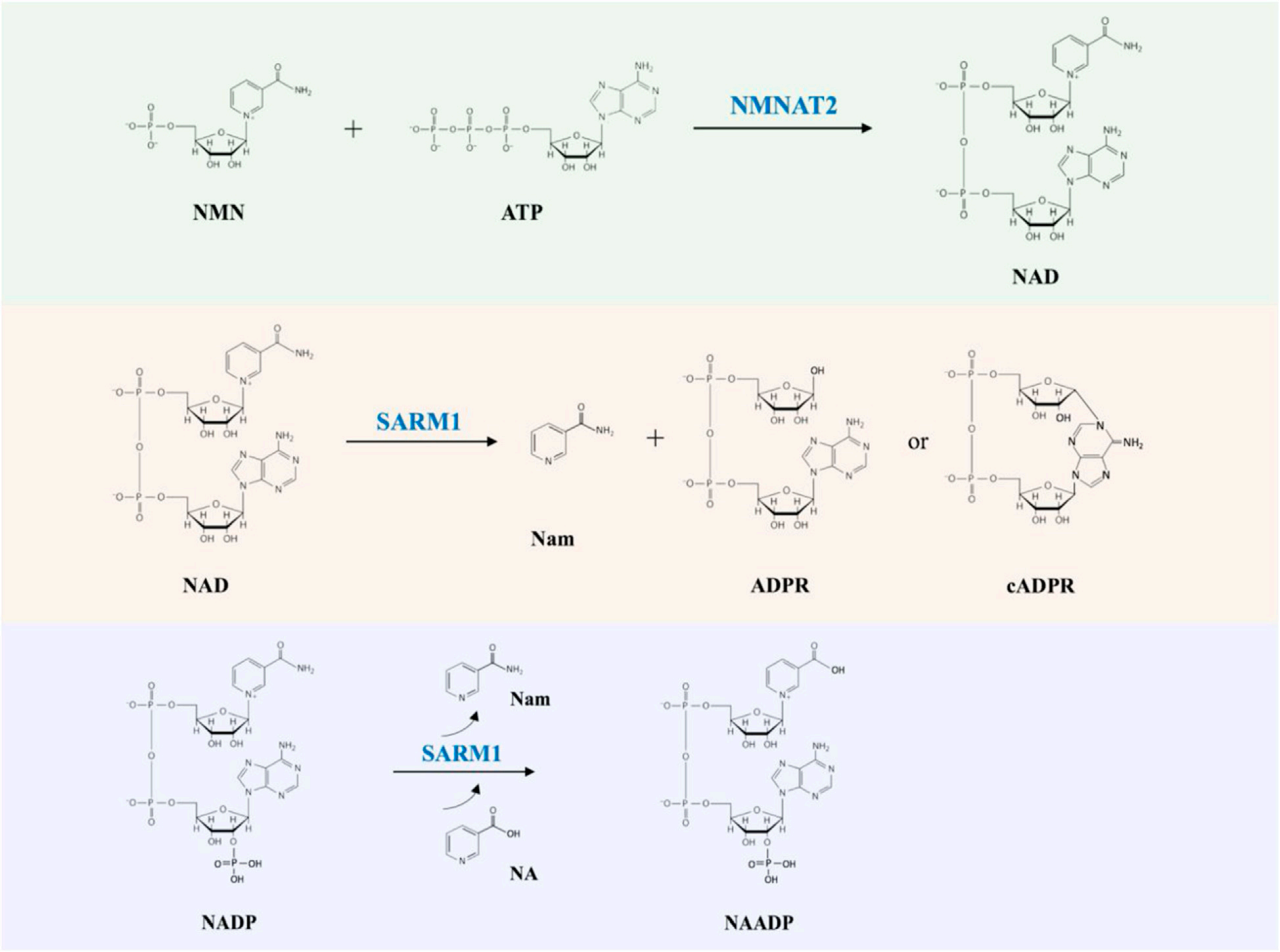
The NMNAT2 and SARM1 catalyzed reactions. NMNAT2 catalyzes the synthesis of NAD from NMN and ATP. SARM1 cleaves NAD into Nam and either ADPR or cADPR; SARM1 catalyzes the exchange of Nam of NADP with NA (nicotinic acid) to produce NAADP (nicotinic acid adenine dinucleotide phosphate).

NAD links two key players in the programmed axon degeneration pathway: the proteins NMNAT2 (nicotinamide mononucleotide adenylyltransferase 2) catalyzing the synthesis of NAD from NMN (nicotinamide mononucleotide) and ATP (adenosine triphosphate), and SARM1 whose multiple activities include hydrolysis of NAD into Nam and ADPR (adenosine diphosphate ribose), cyclization of NAD into cADPR (cyclic ADPR) and base exchange of the nicotinamide group for free bases (Figure 1) (Conforti et al., 2014; Zhao et al., 2019; Coleman and Höke, 2020). The activity of NMNAT2 is essential for axonal survival, while SARM1 activation can kill most cells but is blocked in healthy axons (Gilley and Coleman, 2010; Osterloh et al., 2012; Gerdts et al., 2013; Gilley et al., 2015). NAD also limits SARM1’s activity through blocking its activation (Gilley and Coleman, 2010; Essuman et al., 2017; Jiang et al., 2020; Sporny et al., 2020; Figley et al., 2021). Recently, NMN has been shown to be an activator of SARM1, indicating that NMNAT2 has a dual action in preventing SARM1 activation by converting an activator, NMN, into a molecule that opposes activation, NAD (Di Stefano et al., 2015; Zhao et al., 2019; Bratkowski et al., 2020; Figley et al., 2021).

Intriguingly, SARM1 is not only involved in axon degeneration, but also in innate immunity (Carty and Bowie, 2019). Its structure contains a TIR (Toll/interleukin-1 receptor) domain, which is commonly seen in Toll-like receptors (TLRs) in the innate immune system (O’Neill et al., 2013; Ve et al., 2015). As removing SARM1 delays axon degeneration (Osterloh et al., 2012), it is seen as a good candidate for therapies (Loring and Thompson, 2020). It is currently unclear, though, whether targeting SARM1 could lead to innate immune consequences (Uccellini et al., 2020).

In this review, the mechanisms of axon degeneration are thoroughly examined, while detailing the importance of NAD and related metabolites as signaling molecules. We also discuss whether the role of SARM1 in innate immunity indicates important roles in the response to pathogens and axonal damage.

## Axon Degeneration Research from 1850 to Today

**FIGURE 2:**
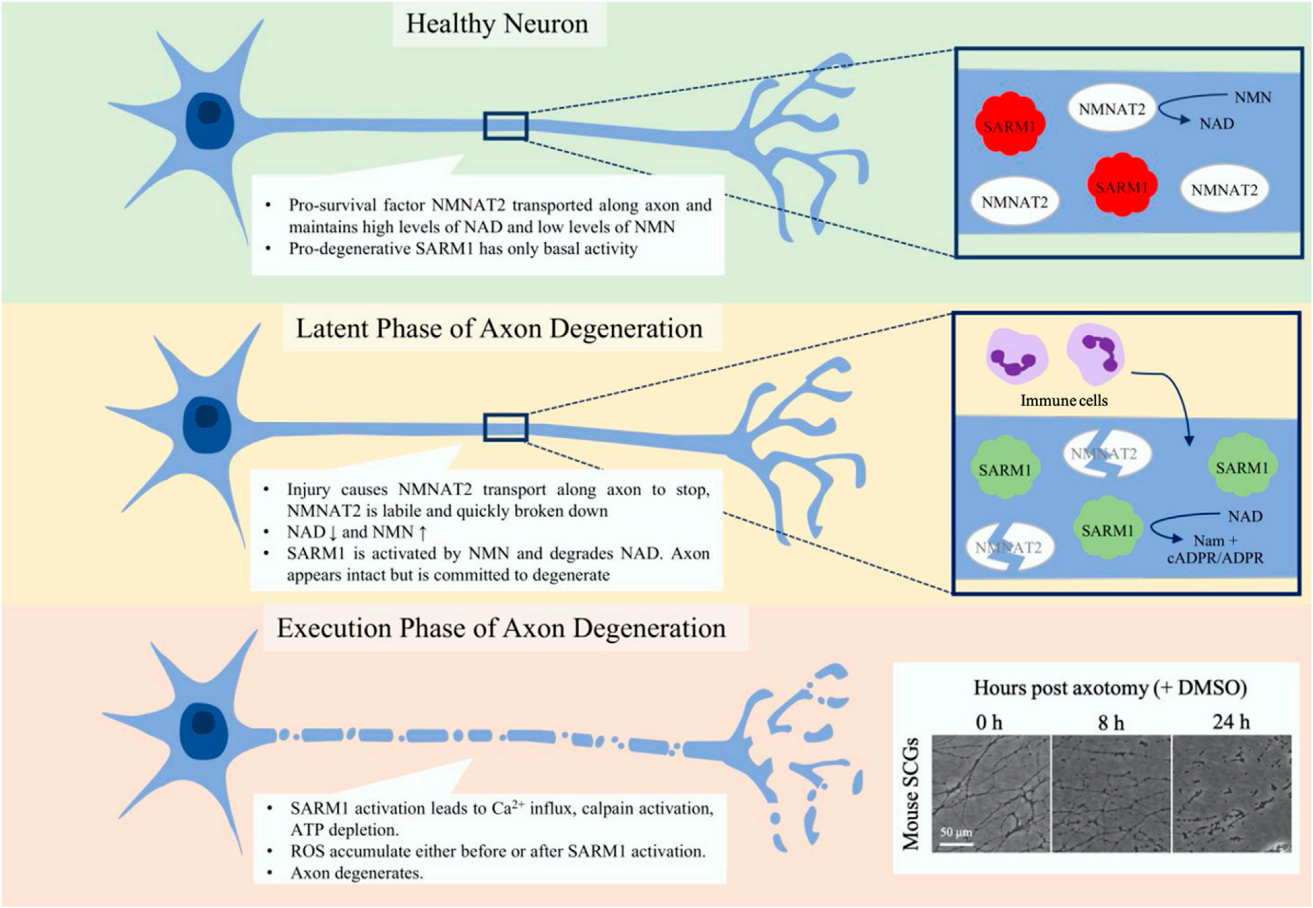
Phases of programmed axon degeneration. Programmed axon degeneration is split into two main phases, the latent phase, where the axons are morphologically normal but are committed to degeneration, and the execution phase, where the axon undergoes gross swelling and granulation as it breaks down. In healthy neurons, NMNAT2 pro-survival factor converts NMN to NAD, maintaining NAD and NMN levels that keep SARM1 pro-degenerative factor inactive. After injury, in the latent phase NMNAT2 starts to be broken down and cannot be transported along the axon. NAD levels thus lower and NMN levels rise, leading to the activation of SARM1. Immune cells are seen to enter the nerve at this stage. After up to 36 h, the axons progress to the execution phase, where Ca^2+^ levels increase, calpains are activated and the axon breaks down. Mouse superior cervical ganglia (SCGs) are often used as an assay for axon degeneration. The box on the bottom corner shows axon degeneration progressing over 24 h post-axotomy. At 8 h axons are committed to degenerate and some have started visibly breaking down. By 24 h, all axons are degenerating and much of what is seen is axon debris.

Axons have been known to degenerate upon injury since Waller’s observations of lesioned nerves of frogs in 1850 (Waller, 1851); however, it was long thought to be a passive process. This process of axon fragmentation is now known as Wallerian degeneration (Figure 2), and differs from neuronal death by apoptosis (Geden and Deshmukh, 2016). While Waller’s work demonstrated the gross degeneration of the distal stump of nerves after transection, it was later work by Ramón y Cajal, in 1928 that detailed the histological changes taking place for the first time (Ramón y Cajal, 1991 English translation).

Axon degeneration can be divided into two phases: the latent phase and the execution phase (Figure 2). The latent phase lasts up to 36 h wherein the axons appear normal and for much of this time retain the ability to conduct an action potential, although the axon becomes committed to degenerate (Tsao et al., 1999; Beirowski et al., 2004; Beirowski et al., 2005; Conforti et al., 2014). This is also the stage in which the immune system is first seen to be involved; tissue-resident macrophages are activated and bone-marrow derived macrophages, neutrophils and phagocytes infiltrate into the nerve (Perkins and Tracey, 2000; Sta et al., 2014; Zigmond and Echevarria, 2019; Boissonnas et al., 2020; Ydens et al., 2020; Chen et al., 2021). There is also an injury-associated increase in the levels of cytokines (Yi et al., 2017). The execution stage becomes apparent once the axon morphology starts to change visibly but molecular execution steps may precede this. The axons fragment and the compound action potential is lost as they become non-functional (Beirowski et al., 2004; Beirowski et al., 2005; Conforti et al., 2014; Sta et al., 2014). Neutrophils may contribute in the early stages of the execution of degeneration by phagocytosing axonal debris (Lindborg et al., 2017), whilst macrophages clear myelin and axonal debris and later are involved in the axon regeneration process (Boissonnas et al., 2020).

Molecules that actively regulate axonal survival and degeneration have long been hypothesized (Lubińska, 1977; Lubińska, 1982). In 1982, Lubińska first proposed that an ‘axonal trophic factor’ is responsible for axon survival and it was likely transported along axons to inhibit Wallerian degeneration, but was unable to identify it (Lubińska, 1982). This unnamed ‘axonal trophic factor’ fits the properties of the protein NMNAT2, and NMNAT2 remains the best candidate for the proposed molecule in Lubińska’s studies (Gilley and Coleman, 2010). Recently, the loss-of-function genetic screens in invertebrates (*Drosophila*) and vertebrates (mouse) identified SARM1, which was shown to be a central executioner in Wallerian degeneration; its deficiency protects severed axons for weeks (Osterloh et al., 2012; Gerdts et al., 2013) and permanently rescues the lethal axon growth deficit of *Nmnat2* null mice (Gilley et al., 2017).

### *Wallerian Degeneration Slow* Mice

The discovery of *Wld* ^*S*^ (*Wallerian degeneration slow*) mice, a strain in which injured axons survive 10-fold longer than in wild-type mice (Lunn et al., 1989), challenged the hypothesis that Wallerian degeneration is a passive process and revolutionized the study of axon degeneration (Coleman and Höke, 2020). The *Wld* ^*S*^ mouse strain arose from a spontaneous intrachromosomal triplication event (Coleman et al., 1998) that results in the formation of a unique chimeric protein, WLD^S^. The WLD^S^ protein was shown to be a fusion of a 70-amino acid N-terminal fragment of ubiquitin ligase UBE4B joined by a unique short linker (Wld18) to NMNAT1 (nicotinamide mononucleotide adenylyltransferase 1) (Conforti et al., 2000; Mack et al., 2001). While NMNAT1 is normally localized to the nucleus of neurons, when fused to UBE4B in WLD^S^, it is partially relocalized to the axons (Babetto et al., 2010). WLD^S^ confers a concentration-dependent axon protection (Mack et al., 2001) and was later found to act by maintaining axonal NMNAT activity if NMNAT2, an axonal protein essential for axon survival, is degraded upon axon injury or is absent for other reasons (Araki et al., 2004; Babetto et al., 2010; Gilley and Coleman, 2010; Gilley et al., 2013; Loreto et al., 2020b). The serendipitous discovery of Wld^S^ mice uncovered one of non-redox roles for NAD and provided the initial evidence that NMNATs and NAD signaling have a role in axon regulation.

## Regulation of Axon Degeneration by Nicotinamide Adenine Dinucleotide-Mediated Signaling

**FIGURE 3:**
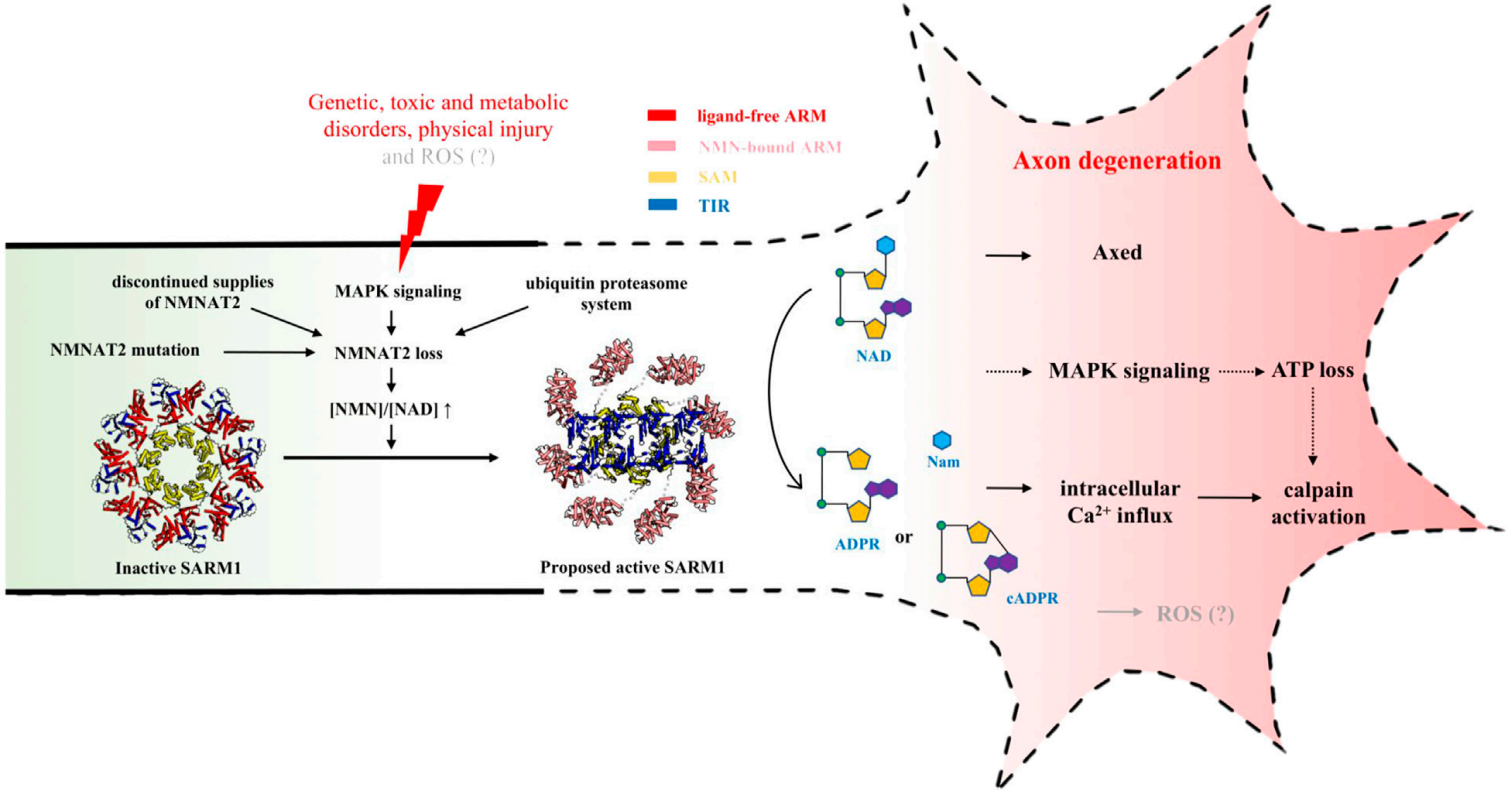
NAD-mediated signaling regulating injury-induced axon degeneration. Upon injury, NMNAT2 loss results in an increased ratio of NMN-to-NAD, which is a signal to activate SARM1 from the inactive state (PDB: 7LD0) to active state (PDB (ARM): 7LCZ; PDB (TIR): 6O0R). Active SARM1 cleaves NAD into Nam and either ADPR or cADPR, initiating the pathways downstream, including intracellular Ca^2+^ influx, ATP loss, Axed activation and eventually axon loss.

In damaged axons and nerves, the level of NAD is known to decrease and the presence of WLD^S^ prevents this decrease suggesting it has an axonal site of action (Wang et al., 2005; Di Stefano et al., 2015), even though it was difficult to detect in axons (Mack et al., 2001). Additional evidence for this came from findings that shifting WLD^S^ or NMNAT1 from nuclei, where these proteins are most abundant, into the cytoplasm (Beirowski et al., 2009; Sasaki et al., 2009a) and subsequently more specifically into axons (Babetto et al., 2010) provided stronger axon protection. Finally, chemical-genetic methods were used to manipulate the stability of axonal WLD^S^ after separating axons from the soma to definitively prove that it has to be present, and stable, in axons to protect them (Cohen et al., 2012; Wang et al., 2015) (Figure 3).

### Nicotinamide Mononucleotide Adenylyltransferase 2

In axons, NAD synthesis relies predominantly on NMNAT2, a labile NMNAT isoform (Gilley and Coleman, 2010). At least in primary neuronal cultures, NMNAT2 appears to be largely synthesized in the cell bodies and transported constantly to axons, where it is required for maintenance of axonal health; axotomy causes its axonal levels to decline rapidly (Gilley and Coleman, 2010). In primary neurons, the majority of NMNAT2 is associated with membranes (Summers et al., 2018), which requires NMNAT2 to be modified by palmitoylation (Milde et al., 2013). Palmitoylated NMNAT2 is relatively unstable, compared to cytosolic non-palmitoylated NMNAT2 (Milde et al., 2013). Depletion of NMNAT2 is sufficient to trigger axon degeneration, which can be counteracted by overexpressing NMNAT2, *Wld* ^*S*^ or other NMNAT isoforms before axons are damaged (Sasaki et al., 2009b; Yahata et al., 2009; Gilley and Coleman, 2010; Yan et al., 2010; Coleman and Höke, 2020), demonstrating its neuroprotective role. Interestingly, protection of axons by NMNAT seems not to depend on an elevated basal level of NAD in axons, but on its enzymatic activity of NAD synthesis after axonal injury (Di Stefano et al., 2015).

CD38 and PARP1 are two important enzymes that consume NAD (Malavasi et al., 2008). In mice, deficiency in CD38 alone, or deficiency in both CD38 and PARP1, leads to an increase of NAD in brain lysates and cultured DRG (dorsal root ganglion) neurons, but axons of these mutated DRG neurons undergo a similar rate of injury-induced degeneration to that seen in the wild-type neurons (Sasaki et al., 2009b).

NMNAT2 is shown to be lost rapidly and spontaneously in damaged neurons as a result of natural turnover before axon degeneration (∼2 h) (Gilley and Coleman, 2010). Axon injury can cause a failure in NMNAT2 transport and so the constant supply of wild-type NMNAT2 into axons is disrupted. The remaining NMNAT2 is regulated by two independent pathways, the MAPK (mitogen-activated protein kinase) signaling pathway and the ubiquitin proteasome system (Milde et al., 2013; Walker et al., 2017; Summers et al., 2018). MAPK signaling promotes the degradation of the palmitoylated NMNAT2 via the MAP3K (MAPK kinase kinase) DLK (dual leucine zipper kinase) and LZK (leucine zipper kinase) (Summers et al., 2018). The non-palmitoylated NMNAT2 is degraded by an E3 ubiquitin ligase complex (Summers et al., 2018). In *Drosophila*, the Highwire (Hiw) E3 ubiquitin ligase has been shown to promote the rapid loss of dNmnat upon injury (Xiong et al., 2012), while its orthologue MYCBP2 (MYC binding protein 2), also known as PHR1 (PAM-Highwire-Rpm-1) E3 ubiquitin ligase promotes NMNAT2 loss in mice (Babetto et al., 2013). A recent serine/threonine-linked ubiquitination mechanism of MYCBP2 suggests ways in which it could be inhibited as a potential therapy in axonal and other disorders (Mabbitt et al., 2020).

NMNAT2 loss is the initial event and an important node in the axon degeneration pathway; thus, understanding the mechanism of NMNAT2 loss and what its loss causes will further our understanding of the axon degeneration process.

### Sterile Alpha and Toll/Interleukin-1 Receptor Motif-Containing 1

Around 2012, loss-of-function genetic screens for axon protection after injury were performed in *Drosophila* olfactory receptor neuron axons (Osterloh et al., 2012) and mouse DRG neurons (Gerdts et al., 2013), both with follow-up *in vivo* studies in mice; both screens identified the protein SARM1, or its *Drosophila* orthologue dSarm, to be involved in the axon degeneration process. Loss of SARM1 provides axonal protection upon injury for weeks *in vivo*, while expression of wild-type SARM1 in *Sarm1* ^*−/−*^ neurons restores the phenotype of rapid injury-induced axon degeneration (Osterloh et al., 2012; Gerdts et al., 2013). Moreover, TIR-1, the SARM1 orthologue in *C. elegans* (*Caenorhabditis elegans*), has recently been shown to inhibit regeneration of damaged motor axons via the NSK-1/ASK1 MAPK pathway, that is independent on its role in axon degeneration (Julian and Byrne, 2020). This dual role of SARM1 in both axon degeneration and regeneration indicates that inhibiting SARM1 can be a way to block axon loss making it a therapeutic candidate for drug development to slow or stop aberrant Wallerian degeneration in several neurodegenerative diseases.

SARM1 has been initially described as one of the adaptor proteins in TLR signaling (O’Neill et al., 2003; Carty et al., 2006). It is a highly conserved protein consisting of an N-terminal ARM (armadillo repeat motif) domain, two central tandem SAM (sterile-alpha motif) domains and a C-terminal TIR domain, responsible for auto-inhibition, oligomerization and NAD hydrolysis, respectively (Mink et al., 2001; O’Neill et al., 2003; Kim et al., 2007; Gerdts et al., 2013; Essuman et al., 2017; Horsefield et al., 2019). In solution, SARM1 exists as ring-shaped octamers, with oligomerization mediated by the SAM domains (Horsefield et al., 2019; Sporny et al., 2019; Bratkowski et al., 2020; Jiang et al., 2020; Sporny et al., 2020; Figley et al., 2021; Shen et al., 2021). In neurons where SARM1 shows the greatest abundance, SARM1 is mostly present in the axons (Osterloh et al., 2012). In healthy axons, SARM1 is held in an inactive state by the auto-inhibitory ARM domains, by means of a direct interaction with and separation of the TIR domains away from each other (Bratkowski et al., 2020; Jiang et al., 2020; Sporny et al., 2020; Figley et al., 2021; Shen et al., 2021). Upon injury and consequent NMNAT2 loss, this auto-inhibition is relieved, permitting the C-terminal TIR domains to self-associate to hydrolyze NAD into Nam, and either ADPR or cADPR, along with other activities, and axons degenerate (Essuman et al., 2017; Horsefield et al., 2019). Although the mechanism of SARM1 activation is still not fully understood, the discovery that SARM1 is a self-regulated NADase (Essuman et al., 2017), together with the discovery of NMNAT2 loss upon injury (Gilley and Coleman, 2010), is particularly important, because it not only solves the puzzle why the activation of SARM1 leads to NAD destruction, but also further provides the evidence for the key role of axonal NAD- and NADP-related metabolism in this death pathway of axon degeneration.

### Nicotinamide Adenine Dinucleotide Depletion vs Nicotinamide Mononucleotide Accumulation Hypotheses

NMNAT2 loss is known to be an event upstream of SARM1 activation (Gilley et al., 2015; Summers et al., 2020). The direct results of NMNAT2 loss are a decrease of the product NAD and an accumulation of the substrate NMN (Di Stefano et al., 2015; Gilley et al., 2015). Therefore, two hypotheses have been proposed to explain the mechanism of the NMNAT2 loss-induced SARM1 activation, the “NAD depletion hypothesis” and the “NMN accumulation hypothesis.”

The “NAD depletion hypothesis” proposes a feed-forward model, where the depletion of NMNAT2’s product NAD is a trigger for SARM1 activation (Gerdts et al., 2016). The studies by Sasaki et al. (2016) found that cytNMNAT1, an engineered NMNAT1 mutant that only targets the cytoplasm and axons, protects axons by blocking the SARM1-mediated NAD depletion, rather than through NAD synthesis. Because NMNAT2 is predominantly expressed in axons, it was inferred that it is likely that NMNAT2 also blocks SARM1-mediated NAD depletion by a similar mechanism in axons. This is consistent with the mouse knockout experiment, in which the embryonically lethal phenotype of *Nmnat2* null mice was rescued by a knockout of SARM1 in the double *Nmnat2* null and *Sarm1* ^*−/−*^ mice (Gilley et al., 2013; Gilley et al., 2015). The recently published cryo-electron microscopy (EM) structures of the NAD-bound SARM1, combined with functional data, provide evidence to support the ‘NAD depletion hypothesis’ (Jiang et al., 2020; Sporny et al., 2020). However, this hypothesis could not explain the effects of modulating NMN described below.

Conforti, Coleman and colleagues proposed an alternative model, in which NMN accumulation upon injury-induced NMNAT2 loss may be a trigger for SARM1 activation in damaged axons (Di Stefano et al., 2015; Di Stefano et al., 2017). To provide support for this hypothesis, they used two independent methods to keep NMN at low levels, with and without altering NAD levels, and studied the effect on axon degeneration. The first method involved the inhibition of NAMPT (nicotinamide phosphoribosyl transferase), an NMN synthesizing enzyme, using the inhibitor FK866 (Sasaki et al., 2009b; Di Stefano et al., 2015); the second method involved exogenous expression of bacterial NMN deamidase, an NMN utilizing enzyme, without altering NAD levels (Di Stefano et al., 2015; Di Stefano et al., 2017). It was found that both methods of blocking the increase of NMN resulted in axon protection, similar to that seen in the *Wld* ^*S*^ phenotype, more modestly with FK866 but as strong as *Wld* ^S^ with NMN deamidase. Further research to study the mechanism demonstrated that NMN indeed accumulated in the distal axons during Wallerian degeneration (Di Stefano et al., 2015; Sasaki et al., 2016) and its accumulation induced the influx of extracellular Ca^2+^, which depended on the appearance of SARM1 and was sufficient to initiate Wallerian degeneration (Loreto et al., 2015). Consistently, fluorescence-based NADase assays *in vitro* illustrated that NMN was indeed an activator of SARM1 (Zhao et al., 2019; Bratkowski et al., 2020; Figley et al., 2021). Moreover, NMN analogues including CZ48, a synthetic cell-permeable molecule (Zhao et al., 2019), and vacor mononucleotide (VMN), a metabolite of neurotoxin vacor (Loreto et al., 2020a), could also activate SARM1 NADase activity, the latter also inducing axon degeneration. However, this hypothesis could also not explain the experimental data that raising the level of both NMN and NAD together protects axons from degeneration (Wang et al., 2015; Sasaki et al., 2016).

Because neither hypothesis could explain all the observations, it was hypothesized that both of NMN and NAD, or NMN-to-NAD ratio, regulate SARM1 activity (Di Stefano et al., 2017). In most cells, the NAD levels are known to be much higher than NMN levels (Formentini et al., 2009; Mori et al., 2014). In the brain, NMN-to-NAD ratio is estimated to be around 0.02 ([NAD]/[NMN] ≈ 49), based on the analysis of the extracts from C57BL/6 mice brain immediately frozen *post mortem* (Mori et al., 2014). Although it is still not clear how much this ratio changes in axons after injury, when NMNAT2 is lost, NMN accumulation and NAD depletion lead to an increased ratio of NMN-to-NAD, which could be a signal for SARM1 activation. Recently, both NMN and NAD were demonstrated to interact directly with the ARM domain of SARM1 (Jiang et al., 2020; Figley et al., 2021). Using cell-, NMR (nuclear magnetic resonance)- and structure-based assays, Figley et al. further demonstrated that SARM1 acts as a metabolic sensor activated by an increasing NMN-to-NAD ratio (Figley et al., 2021). In the proposed model, NAD competes with NMN for the binding site in the regulatory ARM domain, prevents the NMN-induced compaction of this domain and hence promotes the auto-inhibition of the protein (Figure 3) (Figley et al., 2021). Further work is still required to fully understand the mechanism of the relief of auto-inhibition and the activation of NAD-cleavage activity of SARM1.

### Pathways Downstream of Sterile Alpha and Toll/Interleukin-1 Receptor Motif-Containing 1

In 2015, Loreto et al. showed that an increase of intra-axonal Ca^2+^ occurred downstream of SARM1, prior to Wallerian degeneration (Loreto et al., 2015). They argued that this Ca^2+^ increase belongs to the second Ca^2+^ influx after injury, and could be a causative event marking the execution phase of Wallerian degeneration. Active SARM1 cleaves NAD into Nam and either ADPR or cADPR (Essuman et al., 2017; Horsefield et al., 2019), resulting in local NAD destruction and eventually axon degeneration (Gerdts et al., 2015). Both ADPR and cADPR are powerful agents that mobilize Ca^2+^ by targeting the TRPM2 (transient receptor potential melastatin 2) and RyR (ryanodine receptor), respectively (Perraud et al., 2001; Guse, 2004). Consistently, preventing extracellular Ca^2+^ influx using ryanodine receptor antagonist ryanodine confers axonal protection from NMN-induced degeneration (Loreto et al., 2015). One of the possible pathways downstream of SARM1 activation is the activation of neuronal calpains, the Ca^2+^-activated nonlysosomal cysteine proteases that play a role in neurodegenerative events of traumatic brain injury (TBI) (Saatman et al., 2010). It has been shown that calpain activity is increased within minutes in the animal model of TBI (Büki et al., 1999). Consistently, the endogenous calpain inhibitor calpastatin, which inhibits Wallerian degeneration *in vivo* and *in vitro*, is depleted upon physical injury (Yang et al., 2013). Future work is needed to investigate if the change of Ca^2+^ through the SARM1 pathway is necessary and sufficient to activate calpains. SARM1 activation also results in a local energy deficit, inducing pathological axon degeneration. Using traumatic injury as a model, Yang et al. showed that SARM1 is required for the activation of a MAPK cascade involving MEKK4, MLK2, DLK, MKK4, MKK7, JNK1, JNK2, and JNK3, to trigger axon degeneration (Yang et al., 2015). This SARM1-dependent MAPK pathway results in ATP depletion prior to calpain activation and axon destruction, demonstrating that this MAPK cascade is also one of the pathways downstream of SARM1. However, the mechanism of how SARM1 activates the MAPK pathway and how this pathway causes ATP depletion remains obscure. Another possible mediator downstream of SARM1 activation is Axundead (Axed), which was identified in a genetic screen in glutamatergic sensory neurons from the *Drosophila* wing (Neukomm et al., 2017). Axed loss-of-function leads to a complete block of SARM1-induced axon degeneration, suggesting that SARM1 executes its pro-degenerative activity through an Axed-dependent pathway (Neukomm et al., 2017). However, much is still required to be learned regarding to the roles of the Axed orthologues in other species. Finally, there is accumulation of ROS (reactive oxygen species) that occurs either before or after SARM1 activation whose origin and function in axon degeneration requires further study (Press and Milbrandt, 2008; Summers et al., 2014).

Over the past two decades, the NAD-related signaling mechanism associated with the axon degeneration pathway has been slowly being elucidated (Figure 3). It is now known that it starts with the loss of the axon survival factor NMNAT2 through different possible pathways, which results in an increased ratio of NMN-to-NAD due to NMN accumulation and NAD depletion in axons; this leads to the activation of SARM1 NADase activity, local NAD destruction, intracellular Ca^2+^ influx, ATP loss, Axed activation and eventually axon loss, although the potential causative roles of some of the later steps require further clarification. Despite several questions remaining, studying this novel NAD metabolism in axons will enhance our understanding of the degenerative mechanism of axons, and also facilitate the development of drugs for a wide range of neurodegenerative diseases.

## Nicotinamide Adenine Dinucleotide Signaling and the Immune System

### Roles of Toll/Interleukin-1 Receptor Domain-Mediated NADase Activity in the Plant Immune System

SARM1 TIR domain-mediated NAD hydrolysis requires a catalytic glutamate residue at position 642 (E642) in the human protein. An analogous glutamate is also present in plant TIR domain-containing immune receptors, such as *Linum usitatissimum* L6 (E135), *Muscadinia rotundifolia* RUN1 (E100), and *Arabidopsis thaliana* RBA1 (E86) and RPP1 (E164), suggesting there may be NADase activity by the TIR domains involved in plant immune responses; this suggestion has been experimentally confirmed (Horsefield et al., 2019; Wan et al., 2019). In plants, NLRs (nucleotide-binding (NB), leucine-rich repeat (LRR) receptors) detect pathogen effectors and induce immune responses, often characterized by localized cell death (hypersensitive response) to restrict the pathogens to the infection sites (Dangl et al., 2013). Plant NLRs typically consist of an N-terminal TIR domain or coiled-coil (CC) domain, a central NB and oligomerization domain (NOD) and a C-terminal LRR domain, responsible for signaling, oligomerization and recognition of pathogen effectors, respectively (Maekawa et al., 2011; Burdett et al., 2019). Once TIR-NLRs are activated, the central NOD undergoes a conformational change to an oligomerization-prone state, bringing the TIR domains together to facilitate NAD hydrolysis. The recently determined cryo-EM structures of two TIR-NLRs in their active states, *Arabidopsis thaliana* RPP1 and *Nicotiana benthamiana* ROQ1, revealed the protein forms a tetramer, suggesting a minimum functional unit of four TIR domains for NADase activity (Ma et al., 2020; Martin et al., 2020). In both proteins, the TIR domains are arranged through two interfaces, the symmetrical “AE interface,” consisting of the αA helix and the neighboring αE helix, and the asymmetrical “BE interface,” also referred to as the “BB-loop interface” (the BB-loop connects the βB and αB secondary structure elements) (Ma et al., 2020; Martin et al., 2020). Analogous interfaces are also observed in the crystal structure of human SARM1 TIR domain, and mutagenesis showed that the residues mediating these interfaces are important for NADase activity (Horsefield et al., 2019). These similar interactions between the TIR domains illustrate a common mechanism of the TIR domain-mediated NAD cleavage. Interestingly, NAD hydrolysis by plant TIR domains produces a non-canonical variant of cADPR (v-cADPR) (Wan et al., 2019). Future work is required to understand the specificity of the products and the role of this v-cADPR.

### Roles of Toll/Interleukin-1 Receptor Domain-Mediated NADase Activity in the Prokaryotic Immune System

TIR domain-mediated NADase activity is also found in the bacterial and archaeal TIR-domain containing proteins (Tcps), such as *Staphylococcus aureus* TirS, *Echerichia coli* TcpC and *Theionarchaea archaeon* TcpA (Essuman et al., 2018). Like the TIR domains in SARM1 and plant proteins, NAD hydrolysis activity of prokaryotic TIRs also relies on a catalytic glutamate residue (e.g. E216 in TirS, E244 in TcpC and E267 in TcpA). Interestingly, in the available crystal structures of bacterial TIR domains, these glutamates are not found in a location structurally similar to the location in SARM1 and plant TIR domains. Bacterial and archaeal TIR domains from different species also show differences in terms of reaction kinetics and types of the products (Essuman et al., 2018).

In bacteria and archaea, diverse antiphage defense systems have evolved to protect themselves from attack by viruses. Genes encoding these systems are prone to cluster in genomic defense islands (Makarova et al., 2013). A recent study indicated that some bacterial Tcps are found in the defense islands important for anti-phage defense (Doron et al., 2018). The authors named this prokaryotic Tcp defense system as the Thoeris defense system. The Thoeris system is broadly distributed in prokaryotic genomes, encodes an NAD-binding protein ThsA and a TIR domain-containing protein ThsB (Doron et al., 2018). Using *Bacillus subtilis* expressing the Thoeris system (MSX-D12), ThsB has been found to produce a v-cADPR that activates ThsA upon detection of phage infection (Ofir et al., 2021). Activated ThsA further hydrolyzes NAD, causing cell suicide, probably by depleting cellular NAD (Ofir et al., 2021).

In addition, plant TIR domains, SARM1 TIR domain and Tcps also cleave NADP (nicotinamide adenine dinucleotide phosphate) (Horsefield et al., 2019; Wan et al., 2019; Zhao et al., 2019; Essuman et al., 2018), but only SARM1 TIR domain has been shown to have a base-exchange activity (Figure 1) (Zhao et al., 2019). Further studies are required to explain the mechanism of the similar and different functions between different TIR domains.

### Roles of Sterile Alpha and Toll/Interleukin-1 Receptor Motif-Containing 1 in Innate Immunity

The role of NAD metabolism in the immune response has been reviewed in a number of papers (Singhal and Cheng, 2019; Navarro et al., 2021; Navas and Carnero, 2021). It is important to highlight that CD38, predominantly expressed in immune cells, acts as the main enzyme digesting cellular NAD under basal conditions (Camacho-Pereira et al., 2016; Navarro et al., 2021). CD38 has been shown to be involved in NAD cleavage at the inflammation site to maintain T-cell survival (Adriouch et al., 2007). Its expression is increased during infections by a variety of pathogens (Adekambi et al., 2015) and aging (Chini et al., 2020; Covarrubias et al., 2020). CD38 expression is induced by senescence-associated inflammation during aging, which contributes to age-related NAD decline in macrophages (Chini et al., 2020; Covarrubias et al., 2020), as knockout of CD38 prevents this decline and leads to enhanced metabolic health in mice (Camacho-Pereira et al., 2016). Although axon degeneration-related SARM1 NADase activity has been extensively characterized in neurons (rather than immune cells), it still remains unclear if this enzymatic activity plays a role also in the immune system. However, SARM1 has initially been found to be involved in the innate immunity pathways through the ability of its TIR domain to interact with other proteins, rather than its NADase activity (O’Neill et al., 2003; Panneerselvam and Ding, 2015; Carty and Bowie, 2019). TIR domains involved in innate immunity pathways form higher-order assemblies and carry out their signaling function through a mechanism termed signaling by co-operative assembly formation (SCAF) (Nimma et al., 2017; Vajjhala et al., 2017; Nanson et al., 2019).

#### Negative Regulation by Sterile Alpha and Toll/Interleukin-1 Receptor Motif-Containing 1 in Toll-Like Receptor Signaling

TLRs are an important set of germline-encoded pattern-recognition receptors in the innate immune system; they recognize pathogen-associated molecular patterns, including microbial lipids, lipoproteins and nucleic acids (Kawai and Akira, 2010; Thompson et al., 2011). Once activated, TLRs dimerize to create an intracellular TIR-domain signaling scaffold, which then recruits TIR domain-containing adaptor proteins to transfer signals downstream (O’Neill et al., 2013). Six adaptor proteins have been identified in TLR signaling pathways; they are MyD88 (myeloid differentiation primary response gene 88), MAL (MyD88 adaptor-like protein), TRIF (TIR domain-containing adaptor protein-including interferon β), TRAM (TRIF-related adaptor molecule), SARM1 and BCAP (B-cell adaptor for PI3K). Unlike the four key cytosolic adaptor proteins (MyD88, MAL, TRIF and TRAM), SARM1 is not required for TLR signaling, but regulates this signaling through its interaction with other adaptor proteins.

In humans, SARM1 negatively regulates TRIF-dependent TLR3 and TLR4 signaling, hence inactivating the NF-κB (nuclear factor-κB) response (Carty et al., 2006). Using co-immunoprecipitation, yeast two-hybrid assays and GST pull-down assays, it has been shown that SARM1 inhibition of TRIF was achieved through a direct TIR-TIR domain interaction (Carty et al., 2006). Moreover, the BB loop in the SARM1 TIR domain may participate in the interaction, as the BB-loop mutant G601A disrupted SARM1 interaction with TRIF, and its inhibition of the lipopolysaccharide-regulated expression of inflammatory cytokines (Carlsson et al., 2016). The ARM domain plays an inhibitory role, because SARM1 truncation to remove the ARM domain causes a more potent inhibition in TRIF-dependent signaling (Carty et al., 2006). Consistently, in mice, SARM1 expression has been shown to increase during the infection of macrophages with *Burkholderia pseudomallei*, leading to the inhibition of TRIF and hence decreasing IFNβ (β interferon) production (Pudla et al., 2011). In pigs, the SARM1 orthologue negatively regulates NF-κB in a TRIF-dependent pathway during the infection with porcine reproductive and respiratory syndrome virus (Zhou et al., 2013). In horseshoe crabs, *Carcinoscorpius rotundicauda*, the SARM1 orthologue is shown to downregulate TRIF-dependent NF-κB activation during the infection with *Pseudomonas aeruginosa* (Belinda et al., 2008). These results demonstrate the functional conservation of SARM1 as a negative regulator in TRIF-dependent TLR signaling.

Besides inhibiting the NF-κB response, SARM1 also inhibits TRIF- and MyD88-regulated activation of AP1 (activator protein 1) through TIR-TIR domain interactions (Carlsson et al., 2016), or/and direct involvement in inhibiting the phosphorylation of MAPKs via an unknown mechanism (Peng et al., 2010). The ARM domain also self-regulates SARM1 activity in this pathway, as full-length SARM1 is less efficient in this inhibition than the SAM-TIR domains-only-containing fragment. Although it is still not clear if the NADase activity of SARM1 plays any roles in these processes, the ARM domain removal permits self-association of the TIR domains and leads to a more efficient inhibition, suggesting that the SARM1 TIR assembly may facilitate the interaction with the TIR domains of other adaptor proteins, hence likely interfering with the downstream signaling.

#### Up-Regulatory Role of Sterile Alpha and Toll/Interleukin-1 Receptor Motif-Containing 1 in Cytokine and Chemokine Production

SARM1 has also been shown to positively regulate the production of cytokines and chemokines. In the liver of mice suffering from non-alcoholic fatty liver disease (NAFLD) induced by a high fat diet (HFD), SARM1 shows a significant increase in both mRNA and protein levels. SARM1 knockout in these mice leads to a reduction in inflammatory response (a decrease in IL-1β (interleukin-1β), IL-6, TNF-α (tumor necrosis factor α) and MCP-1 (monocyte chemotactic protein-1)) through inactivating TLR4, 7 and 9 signaling, and the NF-κB pathway, supporting a positively regulatory role of SARM1 in cytokine and chemokine production in TLR signaling pathways (Pan and An, 2018). Interestingly, TNF-α also seems to function upstream of SARM1. The Yang lab has recently shown that TNF-α mediates axonal loss via SARM1 in mice liver (Liu et al., 2021), consistent with the finding that TNF-α induces SARM1-dependent axon degeneration in sensory neurons (Ko et al., 2020).

In mice, the neuronal immune response to traumatic axonal injuries was investigated using the *in vivo* model of murine sensory neurons, showing that SARM1 is required for the expression of chemokines *Ccl2*, *Ccl7*, and *Ccl12*, and the cytokine *Csf1*, through a SARM1-JNKs-cJun pathways, and demonstrating an important role of SARM1 in the inflammatory gene expression in the central nervous system (CNS) (Wang et al., 2018). A number of studies were also performed to investigate the physiological role of SARM1 in the CNS against microbial infection, showing that infecting the CNS of *Sarm1* ^*−/−*^ mice with vesicular stomatitis virus (VSV, commonly used as a model for neurotropic viral infection), but not *Listeria monocytogenes*, *Mycobacterium tuberculosis* and influenza virus, restricts viral infection by decreasing cytokine production, suggesting an up-regulatory role of SARM1 in cytokine production (Hou et al., 2013). Interestingly, it has also been shown that *Sarm1* ^*−/−*^ mice were more susceptible to West Nile virus (WNV) infection in a brain-specific manner. Despite a similar reduction of TNF-α observed in *Sarm1* ^*−/−*^ mice, the increased cell death occurred during WNV infection but not VSV infection (Szretter et al., 2009). Although further studies are required to understand this difference, the result indicates that SARM1 contributes to the immune responses during microbial infection in the CNS, linking viral pathogenesis-induced neuronal injury and innate immunity. Because SARM1 NADase activity is required for cell death, it is likely that this enzyme activity also plays a role in this process.

Similar mouse studies were also performed to study the role of SARM1 in the peripheral immune system, by examining the cytokine or chemokine responses to TLR ligands or viral infection in the macrophages of *Sarm1* ^*−/−*^ mice (Gürtler et al., 2014; Uccellini et al., 2020). Using bone marrow-derived macrophages from wild-type and *Sarm1* ^*−/−*^ mice, the Bowie lab demonstrated that SARM1 promotes gene induction of *Ccl5* through recruiting the RNA polymerase II to the *Ccl5* promoter by an unknown mechanism, following TLR4 and TLR7 stimulation (Gürtler et al., 2014). However, the recent data from the García-Sastre lab showed that the deficiency in the production of chemokines, such as *Ccl5*, *Ccl3* and *Ccl4*, was due to the background effects of the knockout strain 129. These chemokine loci are so close to the *Sarm1* gene, that they remain linked to the null mutation in subsequent crosses to C57BL/6. Uccellini et al. (2020) also corrected their previous finding of the important role of SARM1 in VSV infection in the CNS (Hou et al., 2013). These reports indicate a need to re-evaluate the role of SARM1 in the immune responses during microbial infection using methods or resources independent of the 129-derived null strain (Uccellini et al., 2020).

#### Roles of Sterile Alpha and Toll/Interleukin-1 Receptor Motif-Containing 1 Beyond Toll-Like Receptor Signaling

In some species, SARM1 has been reported to have innate immunity functions beyond TLR signaling. Inflammasome activation upon infection and injury result in either cytokine release (without cell death) or pyroptosis (Broz and Dixit, 2016). In mice, SARM1 has been shown to negatively regulate the NLRP3 (NLR family pyrin domain containing 3) inflammasome–dependent caspase-1 activation, hence reducing the release of IL-1β (Carty et al., 2019). On the other hand, SARM1 has also been shown to mediate mitochondrial depolarization (MDP), which is required for optimal pyroptosis and a key to distinguishing from other NLRP3 activators causing cytokine release (Carty et al., 2019). In *C. elegans*, inactivation by RNA interference of TIR-1, the human SARM1 orthologue, decreased worm survival when subjected to fungal and bacterial infections (Carole et al., 2004). This is because TIR-1 is required for the expression of antimicrobial peptide genes (Carole et al., 2004). TIR-1 has also been shown to play a significant role in specifying the asymmetric odorant receptor expression through NSY-1 (ASK1) MAP3K signaling. In this context, TIR-1 functions downstream of a voltage-gated calcium channel and calcium-calmodulin-dependent protein kinase II (CaMKII), UNC-43. It interacts directly with UNC-43 at a high level of Ca^2+^ and localizes NSY-1 to post-synaptic regions of AWC (amphid wing “C”) axons, silencing *str-2* gene (a putative chemoreceptor gene) expression and hence leading to a AWC^OFF^ phenotype (default state) (Chuang and Bargmann, 2005).

## Links to Diseases and Future Therapies

There are few drugs currently on the market that treat neurodegenerative diseases effectively; most either manage the symptoms or slow disease progression, and there are no therapies yet that can reverse the damage done (Durães et al., 2018). This is where research into Wallerian degeneration, in particular SARM1, could prove vital. Wallerian degeneration is a well characterized, druggable pathway that is involved in a number of neurodegenerative diseases (Coleman and Höke, 2020). Wallerian degeneration has been shown to be a cause of axonal death in a number of disease models, including peripheral neuropathies such as those induced by chemotherapy or diabetes (Lapointe et al., 2013; Cashman and Höke, 2015; Geisler et al., 2016; Feldman et al., 2017; Turkiew et al., 2017), traumatic brain injury (Henninger et al., 2016; Ziogas and Koliatsos, 2018), some motor neuron disorders (Ferri et al., 2003; Veriepe et al., 2015; White et al., 2019), Parkinson’s disease (Sajadi et al., 2004; Hasbani and O’Malley, 2006), as well as eye diseases such as glaucoma (Howell et al., 2007; Beirowski et al., 2008; Wang et al., 2013; Zhu et al., 2013; Williams et al., 2017a; Williams et al., 2017b; Fernandes et al., 2018). Loss of SARM1 has been shown to protect mice from peripheral neuropathies (Geisler et al., 2016; Turkiew et al., 2017) and TBI-induced axon loss (Henninger et al., 2016), although there is still a debate on the contribution of SARM1 to ALS (Fogh et al., 2014; Veriepe et al., 2015; Peters et al., 2018).

By targeting SARM1, a drug could be developed that not only slows the progression of one disease, but potentially many that involve axon degeneration. Acute injury, such as chemotherapy-induced peripheral neuropathy (CIPN), is a particularly good initial target for a SARM1 inhibitor, as it is a major reason for limiting the doses of chemotherapies, and the neuropathy onsets only after chemotherapy starts (Cashman and Höke, 2015; Simon and Watkins, 2018; Coleman and Höke, 2020; Bosanac et al., 2021). Ideally, an inhibitor would be given throughout the course of chemotherapy to slow the progression of CIPN or even prophylactically to prevent the neuronal injury from starting.

Another question currently being addressed is whether people have mutations in genes encoding any of the proteins involved in Wallerian degeneration, and if so, do they cause or act as risk factors for neurological diseases? The first evidence of humans with mutations in *NMNAT2* was found in 2019, with biallelic *NMNAT2* loss-of-function mutations found in sisters with polyneuropathy (Huppke et al., 2019). Furthermore, stillborn fetuses with biallelic *NMNAT2* null mutations were also reported (Lukacs et al., 2019). Following earlier human studies implicating this pathway in ALS by GWAS association to the SARM1 chromosomal locus and loss of potential SARM1 regulator STMN2 in human induced pluripotent stem cell (hiPSC)-derived motor neurons (Fogh et al., 2014; van Rheenen et al., 2016; Klim et al., 2019; Melamed et al., 2019), recent work has revealed that human SARM1 variant alleles that hyperactivate SARM1 NADase function and enhance neuronal vulnerability are enriched in patients with sporadic ALS, hereditary spastic paraplegia and other motor nerve disorders (Bloom et al., 2021; Gilley et al., 2021). Whether further mutations to NMNAT2, SARM1 or other proteins involved in the Wallerian degeneration pathway represent risk factors for other neurological diseases in living humans, is yet to be seen. It has been hypothesized that such mutations, affecting the enzymatic activity of NMNAT2 or SARM1, could be considered risk factors for neurodegenerative diseases (Coleman and Höke, 2020).

There are several potential ways to target Wallerian degeneration, but at present these remain in preclinical development. Gene therapy where SARM1 dominant-negative mutants are transfected into neurons using AAV (adeno-associated virus), robustly protected axons from degeneration both in cut DRGs *in vitro* and cut sciatic nerve *in vivo* in mice (Geisler et al., 2019b). Antisense oligonucleotides that target SARM1 and lower SARM1 levels in axons by more than 50% were also shown to slow degeneration *in vitro* (Gould et al., 2021). Furthermore, the first small molecule inhibitors of SARM1 have been discovered and are shown to slow axon degeneration in DRGs to the same level as *Sarm1* ^*−/−*^ (Hughes et al., 2021; Li et al., 2021). Interestingly, the Hughes et al. paper indicated that the inhibitor could be added up to 3 h after injury with the same level of protection, and could even protect axons fated to degenerate through rotenone exposure and promote recovery from the latent stage of degeneration back towards healthier axons (Hughes et al., 2021). Small-molecule inhibitors of SARM1 have recently been tested in hiPSC-derived motor neurons, protecting the axons from degeneration at 16h post-axotomy and *in vivo*, where there was a partial protection of axonal function in mouse models of CIPN (Bosanac et al., 2021; Hughes et al., 2021). These different approaches to tackling axon degeneration and their successes so far in preclinical models are encouraging for the prospects of clinical testing in the near future.

## Conclusions and Future Questions

Axons are eliminated through programmed axon degeneration, a subcellular self-destruction program that can be activated in necroptosis, genetic, toxic metabolic disorders, physical injury, in addition to neuroinflammation. The central executioner of this process is SARM1, an enzyme with NADase, NADPase and base-exchange activities, whose activation is regulated by an upstream NAD synthesizing enzyme, NMNAT2. Emerging structural and functional data for SARM1 indicates allosteric regulation through its N-terminal ARM domain sensing the NMNAT2-mediated change of NMN-to-NAD ratio. Both the regulatory and catalytic sites in SARM1 in the ARM and TIR domains, respectively, represent paths to therapeutic intervention.

SARM1 also plays a role in innate immunity, including regulating TLR signaling, cytokine and chemokine production and gene expression for antimicrobial peptides, and inducing cell death. It is important to be cautious when changing the activity of SARM1, as it may have off-target effects on the immune system of patients. Gilley et al. have demonstrated that *NMNAT2* ^*gtE/gtE*^; *Sarm1* ^*−/−*^ mice live as long as the wild-type ones, so the act of knocking out the proteins themselves does not shorten lifespan (Gilley et al., 2015). However, the mice are kept in specific pathogen-free conditions, so the effects of the changes in the immune response in *Sarm1* ^*−/−*^ mice are currently uncertain. Reassuringly though, the work by Uccellini et al. has shown that many of the supposed immune system effects of SARM1 could be due to passenger mutations in *Sarm1* ^*−/−*^ mouse lines, rather than through SARM1 activity (Uccellini et al., 2020).

Despite the questions surrounding SARM1’s potential involvement in immunity, it remains a promising target for drug development. The possibility of slowing or even halting axon degeneration in a number of neurodegenerative diseases, such as AD, PD, TBI, ALS and peripheral neuropathies, could be incredibly impactful, as these diseases contribute greatly to the global disease burden. Both activators and inhibitors of SARM1 are already known, as is its three-dimensional structure. Basic research continues to investigate how SARM1 is activated and will be crucial in understanding how to inhibit its NADase activity. Excitingly, initial small molecule screens have shown that inhibition of SARM1 is possible and that it does delay axon degeneration *in vitro*. It is likely that further investigation into these initial small molecule inhibitors will move into *in vivo* work and potential new drugs to treat neurodegenerative diseases will be developed. If the rate of discovery in this promising field continues on its current trajectory, it is likely that great strides towards the improvement of human health will be made in the near future–an exciting prospect for all.
